# Supplementary material for: Causal interplay between lactose intolerance and gut microbiota: a combined bidirectional Mendelian randomization and in vivo validation study
Source: Front Nutr. 2026 Jun 1;13:1803337. doi: 10.3389/fnut.2026.1803337 (PMC13265576; doi:10.3389/fnut.2026.1803337)
Supplement: Supplementary file 1 [file Data_Sheet_1.zip › supplementary materials/Forward/leave one out cross validation/ebi-a-GCST90027483.finngen_R12_E4_LACTONAS.pdf]

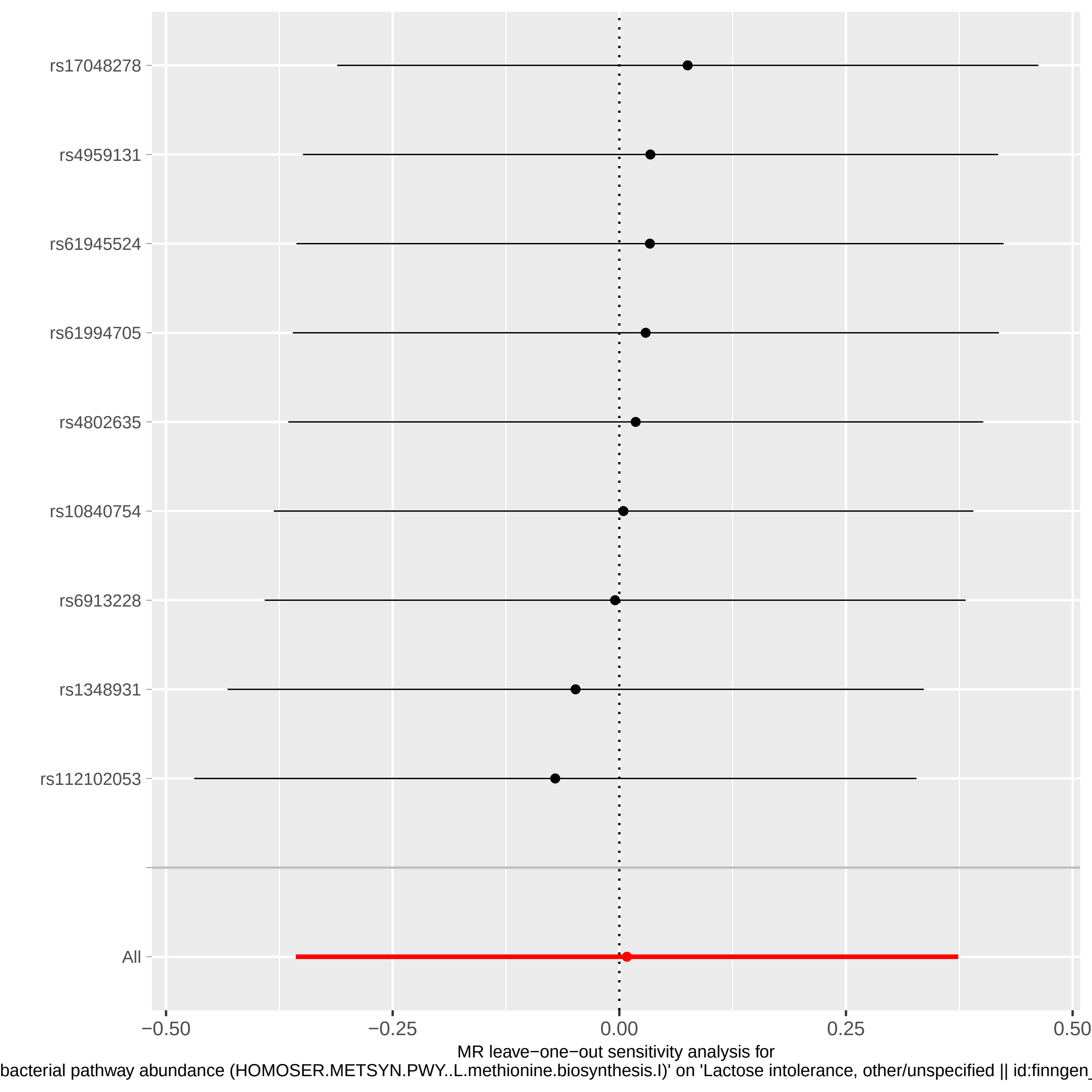
rs17048278
rs4959131
rs61945524
rs61994705
rs4802635
rs10840754
rs6913228
rs1348931
rs112102053
All
−0.50
−0.25
0.00
0.25
0.50
MR leave−one−out sensitivity analysis for
bacterial pathway abundance (HOMOSER.METSYN.PWY..L.methionine.biosynthesis.I)' on 'Lactose intolerance, other/unspecified || id:finngen_
